# Parents’ Perception of the Complementary Baby-Led Weaning Feeding Method: A Validation Study

**DOI:** 10.3390/nursrep10020015

**Published:** 2020-12-01

**Authors:** Elena Martí-Solsona, Víctor M. González-Chordá, Laura Andreu-Pejo, Águeda Cervera-Gasch, Maria Jesús Valero-Chillerón, Desirée Mena-Tudela

**Affiliations:** Nursing Department, Universitat Jaume I, Avda Sos Baynat, 12071 Castellón, Spain; al339152@uji.es (E.M.-S.); pejo@uji.es (L.A.-P.); cerveraa@uji.es (Á.C.-G.); chillero@uji.es (M.J.V.-C.); dmena@uji.es (D.M.-T.)

**Keywords:** baby-led weaning, breastfeeding, complementary feeding, infants, questionnaires

## Abstract

This study aims to construct and validate a questionnaire that allows Parents’ Perceptions of the complementary Baby-Led Weaning feeding method (PaPerc-BLW questionnaire) to be evaluated. An instrumental design was used. Five child nutrition experts took part in the development and content validity. The questionnaire was administered to a sample of 216 Spanish parents of infants aged 0–6 months to determine psychometric properties (construct validity and internal consistency). The factor analysis explained 65.12% of variance with three factors (Promoting infant autonomy and development; Infant’s health; Parents’ fear to apply BLW), and internal consistency was α = 0.67. The mean score of the PaPerc-BLW questionnaire for the total sample was 4.14 (DS = 0.64, 95% CI = 4.06–4.23). Significant differences were found in the parents’ perception of baby-led weaning feeding method based on variables as previous children (*p* ≤ 0.001). Otherwise, the sample included in the study could bias the results, since 98.6% indicated that they were familiar with the BLW method and 62% had previous experience Despite this limitation, the PaPerc-BLW questionnaire offers adequate validity for evaluating parents’ perception of the baby-led weaning method.

## 1. Introduction

The World Health Organization and the United Nations Children’s Fund recommend starting breastfeeding within the first hour after birth and maintain it exclusively for at least the first 6 months of life [1]. After 6 months, as the infant’s nutritional needs increase, it is necessary to introduce other foods to complement breastfeeding without abandoning breastfeeding for at least two years [1]. Therefore, from the age of 6 months onwards, it is time to introduce other foods into the infant’s diet, understood also as complementary feeding.

Complementary feeding is understood as a process in which solid or liquid food is offered to complement breastfeeding [1]. Traditional complementary feeding has been recommended in the form of mashes taken with a spoon that combine one food or more with milk, broth or water [2]. However, currently, there is evidence for choosing alternative methods like Baby-Led Weaning (BLW). This method consists of offering infants food without crushing or grinding to self-manage infant intake [3], using their own tools, such as their hands.

To initiate complementary feeding, the infant’s age is normally used as a reference. However, to initiate the BLW method, babies’ neurological development should allow them to remain seated, coordinate hand movements and to no longer exhibit the extrusion reflex. By the BLW method, babies decide what foods to take, how much and at what rate, in which thus confers the babies a more active role and participation in family meals [4], facilitating the use of family meals. The BLW method is, therefore, an on-demand diet responsive feeding during structured meal times and can allow the babies better control of satiety [5]. It would be positive if babies could tell by gestures when they no longer want to eat to avoid forced feeding, not only with BLW, but also if they are spoon-fed.

The BLW method provides benefits, but also inconveniences. Advantages include promoting exclusive breastfeeding up to the age of 6 months [6] to gain healthy eating habits, and to achieve better transition to solid feeding [7]; better infant psychomotor development [8]; obesity prevention [9]; consistent zinc intake [10]. Some highlighted inconveniences include risk of asphyxia when a series of safety norms are not met [9], nutritional caloric imbalance [5] or iron deficiency [11], both related to the parent’s lack of knowledge about adequate nutrition. However, when BLW has been analyzed in contexts in which parents have been previously presented with basic information, no differences were found in nutritional status, nutrients intake or choking [5,10,12].

The scarce information available about this method means that parents do not know this alternative way to introduce solids and may be skeptical or even worried when used it. In fact, a recent study conducted in Spain shows that only 38% of the sample had heard of BLW and the overall prevalence of BLW was estimated at 14% [13]. Knowing the causes that concern parents and limit the use of this method can help health professionals, specifically nursing and midwifery, to give better information, advice and professional support during this stage of the baby’s development. In the literature, there are validated questionnaires to assess feeding styles in young children [14] or the appetite of babies during the period of exclusive milk-feeding [15]. However, no questionnaires were found to measure parents’ perception of baby led weaning or other methods to introduce new foods into the babies’ diet. Thus, this study aims to construct and validate an instrument that allows parents’ perceptions of the BLW complementary feeding method to be assessed (PaPerc-BLW).

## 2. Materials and Methods

An instrumental design was used to develop and validate the PaPerc-BLW questionnaire according to literature recommendations [16]. The study was conducted between November 2018 and March 2019.

### 2.1. Questionnaire Design and Content Validity

A literature review was firstly conducted to confirm that there were no similar instruments. This review served to substantiate an initial questionnaire version. Subsequently, five health professionals with experience in nutrition were invited to participate in a nominal group technique to establish the content validity of the instrument, in accordance with previous studies [17]. In addition, a mother with previous experience in performing BLW was included in this group, providing a closer view of reality. A cognitive pretest was carried out as a method to determine if the questionnaire was working correctly and if there were elements that were difficult to understand [18]. To do this, a member of the research team administered the questionnaire to a sample of 25 participants, with the same characteristics as the target population, and later interviewed them to assess the difficulty of the items, the response scale and the general appearance of the questionnaire.

The final questionnaire consisted of 17 items that measured parents’ perception of BLW on a 5-point Likert scale from 1 (Totally disagree) to 5 (Totally agree). In this way, a higher score on the PaPerc-BLW questionnaire suggests that parents have a better perception of the BLW method and would be willing to use this method.

### 2.2. Psychometric Properties

The questionnaire was administered to a Spanish sample of 251 mothers and fathers of infants aged between 0–6 months to determine the psychometric properties of the questionnaire (construct validity and internal consistency). Parents with babies in this age group were included because they may be more interested in and have more information about complementary feeding methods, as they will soon make the decision on which method to use. The sample size was established at between 5 and 10 subjects per item (17 items) [19]. Data collection took place in groups related to maternity and breastfeeding (Mamare, Amamanta, and Pit I Prou) in Facebook, Twitter, or WhatsApp for a 10-day period, according to literature recommendations [20].

In addition, the questionnaire included a brief description of the BLW method. Age, gender (Male; Female), academic degree (Doctorate or students; Bachelor/Master; Diploma/Degree; Senior technician; Mid-level technician; Graduated), previous children (No children; One child; Two children; Three children or more) were collected as socio-demographic variables. Moreover, the questionnaire included the variable type of milk feeding (Maternal/Formula/Mixed) and variables about the parents’ experience with BLW: (i) Familiar with BLW (Yes/No); (ii) Previous experience with BLW (Yes, No); (iii) Willingness to apply BLW (Yes/No/Do not know).

### 2.3. Statistical Analysis

A descriptive analysis was carried out depending on the nature of the variables. After studying the normality and homoscedasticity, a bivariate analysis was performed with U Mann–Whitney or Kruskall–Wallis tests. Construct validity was calculated by the Kaiser–Meyer–Olkin (KMO) test and Bartlett’s test of sphericity, in which resulted in an exploratory factor analysis through extracting factors using principal components with varimax rotation. A minimum factor load of 0.4 was established as a threshold to retain an item within a certain factor [21]. If an item had loads greater than 0.4 in different factors, the research team decided by consensus on which factor to retain the item, considering the factor load and the coherence between the rest of the items and the possible meaning of the dimension. The instrument’s internal consistency was calculated with Cronbach’s alpha. Analyses were performed with SPSS and the significance level was *p* ≤ 0.05.

### 2.4. Ethical Considerations

The study was designed in accordance with the principles of the Declaration of Helsinki (charity, no maleficence, autonomy and justice) and with Spanish Law 03/2018 on Protection of Personal Data and Guarantee of Digital Rights Organic. The study was approved by the Deontological Commission of the Universitat Jaume I (CD/28/2020). No personal data, IP address or email that could compromise the participants’ identity were collected, and answering the questionnaire implied giving consent. Participants were informed of these aspects before voluntarily answering the questionnaire.

## 3. Results

### 3.1. Sample Description

Of the whole sample, 99.1% (*n* = 214) were women whose mean age was 34.12 years (SD = 4.07), 49.1% (*n* = 106) had one child, and 71.7% (*n* = 155) had completed university studies. Moreover, 87.5% (*n* = 189) fed their babies exclusively by breastfeeding, and 98.6% (*n* = 213) had heard of the BLW method, while 62% (*n* = 134) had previously practiced BLW. The socio-demographic data are found in Table 1.

### 3.2. Content Validity and Psychometric Properties of the PaPerc-BLW Questionnaire

Experts reached a consensus during the face-to-face meeting held about item adequacy. Minor modifications were made to some items and experts decided to add item 15 (see Table 1). The pilot test (*n* = 25) confirmed that the questionnaire was understood and easy to complete. The mean completion time was 5 min.

To evaluate construct validity, 251 responses were collected, of which 13.94% (*n* = 35) were eliminated because answers to questions were incomplete. The viability of the factor analysis was confirmed by the Kaiser–Meyer–Olkin test (*p* = 0.907) and Bartlett’s test of sphericity (X^2^ = 2655.2; *p* ≤ 0.001). The factor analysis explained 65.12% of variance through three factors (factor 1 = 42.79%; factor 2 = 13.23%; factor 3 = 9.09%). The factorial structure showed adequate internal consistency (Cronbach’s Alpha = 0.67) (Table 2).

### 3.3. Perception of Baby-Led Weaning

The mean score of the PaPerc BLW questionnaire for the total sample was 4.14 (DS = 0.64, 95% CI = 4.06–4.23). The total score of the PaPerc-BLW questionnaire only showed significant differences depending on the number of children, so that the higher the number of children, lower scores were obtained (No children: m = 4.22, DS = 0.66; One child: m = 4.17, DS = 0.6; Two children m = 3.81, DS = 0.69; Three or more children m = 3.71, DS = 0.463) (*p* = 0.043). However, no significant differences were found in the global questionnaire score depending on whether (m = 4.09; DS = 0.611) or not (m = 4.24; DS = 0.685) the parents had previous experience in using the BLW method (*p* = 0.509). The other variables did not offer significant differences either. Table 3 shows the descriptive results obtained for the global sample, each dimension and each item.

Otherwise, the Infant’s autonomy and development dimension obtained a mean score of 4.53 (DS = 0.83; 95% CI = 4.43–4.65). Parents with a child (m = 4.77; sd = 0.515; 95% CI = 4.33–4.69) and without children (m = 4.515; DS = 0.839; 95% CI = 4.33–4.69) obtained significantly higher scores than parents with two children (m = 4.12; sd = 1.18, 95% CI = 3.61–4.63) and with three or more children (m = 2.93; sd = 2.1; 95% CI = 0.49–6.28) int his dimension (*p* = 0.021). In addition, there were significant differences depending on whether type of milk was maternal (m = 4.57; sd = 0.81; 95% CI = 4.12–5.19), formula (m = 4.61; sd = 0.77; 95% CI = 4.12–5.19) or mixed (m = 4.09; sd = 1.02, 95% CI = 3.52–4.65) (*p* = 0.017). Otherwise, parents who were willing to use this method (m = 4.63; sd = 0.74; 95% CI = 4.53–4.74) obtained a significantly higher score than parents with doubts (m = 4.25; sd = 0.54, 95% CI = 3.88–4.61) or those who would not use it (m = 2.97, sd = 1.05, 95% CI = 2.22–3.72) (*p* < 0.001).

The Fear of the BLW dimension obtained a mean score of 2.19 (DS = 0.75; 95% CI = 2.08–2.29). Parents who were hesitant to use this method (m = 3.53; sd = 0.52; 95% CI = 3.17–3.88) obtained significantly higher scores than parents who did not want to use it (m = 2.94, sd = 0.74; 95% CI = 2.49–3.48) and those parents who stated that they would use it (m = 2.07; sd = 0.65; 95% CI = 1.97–2.16) (*p* < 0.001).

Finally, the infant’s health dimension obtained the lowest mean score (m = 1.57; DS = 0.87; 95% CI = 1.45–1.69). Parents willing to use this method (m = 1.52; sd = 0.84; 95% CI = 1.41–1.64) obtained significantly lower scores than parents who would not use it (1.75, sd = 0.82; 95% CI = 1.15–2.34) or those who had doubts (m = 2.18, sd = 1.1; 95% CI = 1.44–2.92) (*p* = 0.025).

## 4. Discussion

The present study validated the PaPerc-BLW questionnaire, which assesses parents’ perception of the BLW method. On the on hand, the factor analysis explained 65.12% of variance with three factors: (i) Promoting infant autonomy and development; (ii) Infant’s health and (iii) Parents’ fear to apply BLW. For the Factor 1 the main benefits highlighted in the literature for this feeding method are the infant’s autonomy and development [8]. For Factor 2, the BLW method has been shown to have a significant impact on child health [22], for example, with childhood obesity [23]. The last factor, fear of establishing BLW, can become a determining factor when implementing this feeding method. This factor can be represented in the international literature as concern [24] or lack of trust from parents [2,25]. Thus, these factors can be considered representative of complementary feeding with the BLW method. However, it should be mentioned that some items had factor loadings greater than 0.4 points in various factors. The research team decided to maintain this structure for consistency with the literature and the results of the explained variance. On the other hand, the instrument’s internal consistency was acceptable. However, the results of Cronbach’s alpha showed an internal consistency that can be considered low in “The infant’s health” dimension. This dimension consisted of two items that reached the cut-off point for factor load only in this dimension. For this reason, despite the results of internal consistency, the research team decided to keep the dimension and the items, hoping to verify the results with larger samples and a confirmatory factor analysis in future studies. Male participation in the sample was low, perhaps because they are less involved in their baby’s upbringing because this has traditionally been a maternal role [26]. It is important to assess this aspect as fathers’ active participation in the parenting process is related to socio-health benefits for both the infant and family [27]. A more in-depth analysis of this aspect could be performed, but is beyond the scope of this study [26].

The percentage of mothers who breastfed their babies (87.5%) was much higher than the national data reported by the National Institute of Statistics (Spain). Perhaps this data should reveal a greater interest in BLW on the part of women who breastfeed or women who choose to breastfeed consider BLW as a more natural method that promotes the autonomy of their baby when introducing solid foods. However, the results of this study do not support this hypothesis and subsequent studies with larger samples should confirm these assumptions. It is noteworthy that nutritional experiences during childhood include developing future healthy eating habits [28].

Regarding parents’ perception, the majority of the sample was familiar with BLW but some parents still expressed their fears of offering their children food via the BLW method, such as fear of choking or asphyxia, nutritional deficiency or lack of energy [7] or iron deficiency and impaired growth [9]. Notably the portion of offered food with this feeding type is important because it will affect how the infant manages food. It has been evidenced that larger pieces are avoided and babies better accept food with different textures by the BLW method [29]. It is necessary for health professionals to continue working on this issue as some studies show a relation between education and improving child health [30,31] and, specifically, improvement in complementary feeding practices in relation to parental education [32]. It is also important to recognize the need for training health professionals in the BLW supplementary feeding method as other studies demonstrate that only 21% of participants obtained counselling from a professional in the health field [33].

Among the main limitations found, the sample included in the study could bias the results since 98.6% indicated that they were familiar with the BLW method and 62% had previous experience. However, we did not specifically look for experienced parents. Thus, it is possible that the BLW method is not common in other contexts, but it seems that in our setting the prevalence is high and it may be due to the fact that this topic is included in the prepartum talks. Future studies should be carried out in populations, where the prevalence of BLW is not so high to advance the validation of this questionnaire. Moreover, the sample was obtained over an online platform, so some mothers or fathers may have decided to not respond because they were not interested in the method, in which would limit the quantity and variety of responses. Another limitation caused by the type of platform used was the impossibility of performing a temporal stability test. Indeed, it is very difficult for the same sample to assess such temporal stability when a test is sent via social networks. Furthermore, achieving an acceptable internal consistency is noteworthy, although the dimension “The infant’s health” may improve in this regard. This may be related to conducting a single round of content validity with experts and it is worth noting the small sample size in the initial pilot test and its possible bias, because the instrument was launched through breastfeeding groups. For these reasons, the results of this study on parents’ perception of BLW are difficult to generalize and should be viewed with caution. Otherwise, the validation results of the PaPerc-BLW questionnaire are considered relevant, since no similar questionnaires were found in the literature review. A change in strategy for future research is proposed to obtain a more representative sample and to determine temporal stability and to validate the questionnaire through a confirmatory factor analysis. In addition, completing the validation of this questionnaire opens new research lines aimed at identifying the reasons why parents do not use BLW as a complementary feeding method or developing and evaluating specific strategies and interventions to improve their perception of this method.

## 5. Conclusions

In conclusion, the PaPerc-BLW questionnaire is a valid reliable instrument to evaluate parents’ perceptions of the BLW complementary feeding method. The instrument consists of 17 items measured on a Likert scale organized on three dimensions: (i) Infant’s autonomy and development; (ii) Infant’s health; (iii) Parental fear to implement this method. Some statistically significant relationships appear between the variables number of children, type of breastfeeding and willingness to use BLW and the results of the PaPerc-BLW questionnaire, opening new lines of research on parental advice. It is also necessary to continue with the questionnaire validation process with larger samples and to administer it in other contexts than the Spanish one.

## Figures and Tables

**Table 1:** Sample description.

	%	*n*
**Gender**		
Female	99.1	214
Male	0.9	2
**Previous children**		
No children	38.4	83
One child	49.1	106
Two children	10.6	23
Three children or more	1.9	4
**Academic qualifications**		
Doctorate or students	5.6	12
Bachelor/Master	41.7	90
Diploma/Degree	24.5	57
Senior technician	15.3	33
Mid-level technician	7.4	16
Graduated	5.6	12
**Type of milk feeding**		
Maternal	87.5	189
Formula	5.6	12
Mixed	6.9	15
**Familiar with BLW**		
Yes	98.6	213
No	1.4	3
**Previous experience with BLW**		
Yes	62	134
No	38	82
**Willingness to apply BLW**		
Yes	90.3	195
No	5.1	11
I do not know	4.6	10

**Table 2:** Matrix of components and internal consistency of the PaPerc-BLW questionnaire.

Dimensions and Item	Factors			α
	1	2	3	
**Infant autonomy and development**				0.959
1. BLW facilitates the transition to family feeding.	**0.936**	0.118	0.042	0.628
2. BLW makes it easier for the baby to adapt to new flavours and consistencies.	**0.920**	0.137	0.049	0.628
3. BLW promotes the baby’s autonomy.	**0.906**	0.231	0.026	0.623
4. BLW promotes chewing.	**0.896**	0.231	0.026	0.630
5. BLW promotes the development of fine motor skills.	**0.891**	0.216	0.078	0.625
6. BLW promotes the baby’s maturational development.	**0.892**	0.140	0.067	0.626
7. BLW prevents obesity.	**0.756**	0.039	0.042	0.642
8. BLW favours varied food intake by the baby	**0.797**	0.145	0.184	0.629
**The infant’s health**				0.583
9. When the method starts, no milk supplementation is necessary ^a^.	−0.220	0.194	**0.713**	0.679
10. With BLW, all food types can be given without exception.	−0.208	0.275	**0.677**	0.678
**Fear of BLW**				0.686
11. Babies do not gain enough weight with this method.	−0.461	**0.465**	0.391	0.680
12. BLW frequently causes nutritional deficiencies.	−0.561	**0.430**	0.157	0.681
13. Performing BLW involves an increased risk of choking or asphyxia.	−0.564	**0.441**	−0.024	0.697
14. I am afraid of practicing this method with my baby.	−0.368	**0.551**	−0.217	0.682
15. I would apply this method only if I were present.	0.137	**0.613**	−0.381	0.637
16. My work prevents me from sharing meals with my baby; therefore, I favour using traditional methods (mashes), not BLW.	−0.197	**0.547**	−0.349	0.671
17. Further training could help me change my mind about the BLW method.	0.043	**0.612**	−0.164	0.645

^a^ Item in reverse. Bold indicates the dimension to which each item belongs.

**Table 3:** Descriptive results of the PaPerc-BLW questionnaire.

Dimensions and Items	m	DS	95% CI
**Infant’s autonomy and development**	4.53	0.83	4.43–4.65
** 1**	4.61	0.91	4.48–4.73
** 2**	4.65	0.88	4.53–4.77
** 3**	4.71	0.82	4.60–4.82
** 4**	4.62	0.95	4.49–4.75
** 5**	4.71	0.78	4.60–4.81
** 6**	4.47	0.97	4.34–4.60
** 7**	4.15	1.12	4.00–4.30
** 8**	4.40	1.02	4.27–4.54
**Infant’s health**	1.57	0.87	1.45–1.69
** 9 ^a^**	1.45	9.49	1.32–1.58
** 10**	1.69	1.11	1.54–1.84
**Fear of BLW**	2.19	0.75	2.08–2.29
** 11**	1.53	1.04	1.39–1.67
** 12**	1.41	0.808	1.30–1.52
** 13**	1.81	1.16	1.66–1.97
** 14**	1.94	1.34	1.76–2.12
** 15**	3.49	1.39	3.30–3.67
** 16**	2.12	1.35	1.94–2.30
** 17**	3.00	1.63	2.78–3.21
**Global PaPerc-BLW**	4.14	0.64	4.06–4.23

^a^ Item in reverse.
